# Artesunate prevents knee intraarticular adhesion via PRKR-like ER kinase (PERK) signal pathway

**DOI:** 10.1186/s13018-019-1445-x

**Published:** 2019-12-17

**Authors:** Hui Chen, Jin Tao, Jingcheng Wang, Lianqi Yan

**Affiliations:** 1 Dalian Medical University, Dalian, Liaoning People’s Republic of China; 2 Department of Orthopedics, Clinical Medical College of Yangzhou University, Subei People’s Hospital of Jiangsu Province, Yangzhou, People’s Republic of China

**Keywords:** Artesunate (ART), PRKR-like ER kinase (PERK), Apoptosis, Intraarticular scar adhesion

## Abstract

**Background:**

Intraarticular scar adhesion refers to a serious complication caused by knee surgery or trauma, leading to various sequelae (e.g., articular cartilage degeneration and knee joint stiffness). Artesunate (ART) has exhibited an effect to suppress fibroblast proliferation, whereas the exact mechanism remains unclear. This study aims to delve into the possible mechanism of ART in suppressing joint adhesion.

**Methods:**

The effect of ART on reduced intraarticular adhesions was ascertained by histological staining and immunohistochemical analysis through vivo experiments. Cell Counting Kit-8 (CCK-8) assay, Western blot analysis, flow cytometry, and tunnel staining were used to detect the effect of ART in promoting fibroblast apoptosis and delve into its possible signaling pathway.

**Results:**

The results of hematoxylin-eosin (HE) staining suggested that the number of fibroblasts decreased with the increase in ART concentration. The results of Masson staining were similar, with the increase in concentration, the collagen content decreased. Immunohistochemical results showed that the expression of endoplasmic reticulum stress (ERS) characteristic proteins 78 kDa glucose-regulated protein 78 (GRP78) and C/EBP homologous protein (CHOP) increased in a concentration-dependent manner. CCK-8 results suggested that ART could inhibit fibroblast viability in a concentration- and time-dependent manner. Results of flow cytometry, tunnel staining, and Western blot suggested the apoptosis of fibroblasts occurred after ART treatment. Cells with caspase inhibitors were treated, and apoptotic proteins cleaved-poly ADP-ribose polymerase (cleaved PARP) and cleaved-caspase 3 were detected; the results showed that the apoptotic effect of ART was reduced. The expressions of ERS-related protein CHOP and apoptosis-related protein Bax were upregulated, while the expression of Bcl-2 was downregulated, and the ratio of Bax/Bcl-2 increased in a concentration-dependent manner. Continuous detection of PRKR-like ER kinase (PERK) pathway-related proteins showed that the expression of p-PERK and phosphorylating eukaryotic initiation factor 2α (p-eIF2α) increased in a time-dependent and concentration-dependent manner. PERK pathway inhibitors could partially inhibit ART-mediated apoptosis through PERK pathway.

**Conclusions:**

ART can promote fibroblast apoptosis through PERK pathway, a classical ERS pathway, and thus prevent fibrosis in the surgical area after joint surgery.

## Introduction

The main weight-bearing joint, the knee joint, is prone to injury and degenerative lesions for its high mobility and lack of soft tissue protection. Intraarticular scar adhesion of knee joint refers to a common complication after surgery or trauma, and it critically affects the prognosis of surgery. It has been one of the difficult problems facing the world’s orthopedic department. It often causes complications (e.g., the limited range of motion of joints, chronic joint pain, and even degenerative diseases of articular cartilage) [1]. Accordingly, a way is required to address this problem in the clinic.

Numerous ways have been developed to mitigate intraarticular adhesion after knee surgery or trauma. For instance, some scholars studied the efficacy of manual lysis in the treatment of knee joint adhesions under anesthesia. Their results suggested that this method exhibits significant advantages in the treatment of early knee joint adhesion [2]. Others also proposed to use drugs (e.g., the use of mechanical barrier drugs, anti-inflammatory drugs, inhibition of fibroblasts drugs) to prevent knee adhesions and have achieved certain results in basic experiments [3–6]. However clinically, there is still controversy about the feasibility of drug-induced knee adhesions, so new drugs which have small side effects to solve this problem are required.

Artesunate (ART), i.e., artesunatum, is one of the artemisinin derivatives that exhibit a sesquiterpene structure. It is a fast-acting, low-toxic antimalarial drug used for the treatment of malaria parasite infection in adults, children, and pregnant women, having saved millions of lives [7, 8]. Its biological activity and metabolites have been widely discussed, including anti-inflammatory, anti-sepsis, anti-tumor, radio-sensitization, antibacterial-sensitization, etc. [9–12].

ART, an antimalarial drug, also exhibits certain anti-fibrotic effects (e.g., pulmonary fibrosis, liver fibrosis, and renal fibrosis) [13–15]. ART can significantly downregulate the expression of TGF-β1 and TNF-α in rats with pulmonary fibrosis, thereby mitigating the pulmonary fibrosis. It also can prevent oxidative stress, suppress proinflammatory cytokines, inhibit hepatic stellate cell proliferation, and induce hepatic stellate apoptosis, thus affecting the synthesis and metabolism of extracellular matrix and exerting anti-hepatic fibrosis [16]. Accordingly, we assumed that ART can also suppress fibroblast proliferation, thereby mitigating postoperative adhesions in the knee joint.

The endoplasmic reticulum (ER) is a vital organelle involved in protein synthesis, modification and processing, as well as the folding, the assembly, and the transport of nascent peptide chains. The classical mechanisms of endoplasmic reticulum stress (ERS)-mediated apoptosis are primarily classified into two types (unfolded protein response and calcium signaling) [17]. On the one hand, once unfolded or misfolded proteins are assembled, the unfolded protein response (UPR) will serve as a protective mechanism to protect cells from being harmed. However, if the unfolded protein or the misfolded protein is not eliminated, it may cause apoptosis [18]. On the other hand, the calcium homeostasis of the endoplasmic reticulum of the cell is also crucial to the cell. Once the calcium homeostasis is broken, the apoptosis will be induced. In recent years, it has been reported that ERS-mediated apoptosis is conducive to the treatment of fibrotic diseases. PRKR-like ER kinase (PERK) suppresses protein synthesis in cells by phosphorylating eukaryotic initiation factor 2α (eIF2α), and it upregulates C/EBP homologous protein (CHOP) gene by activating transcriptional activator 4. The expression of activated apoptotic pathways is involved in the cellular stress response process, and the PERK pathway is one of the critical pathways for ERS. It has recently been reported that ERS-mediated apoptosis is capable of treating fibrotic diseases [19].

In the present study, we aimed to investigate whether ART can induce fibroblast apoptosis through PERK pathway, reduce, or even prevent intraarticular adhesion after knee surgery or trauma. We considered it an effective method to mitigate knee joint adhesion.

## Materials and methods

This experimental study was approved by the Ethics Committee and Research Committee of Jiangsu Subei People’s Hospital. Primary human fibroblast cell line was provided by GuangZhou Jenino Biotech Co., Ltd. Fibroblasts were cultured in Dulbecco’s modified Eagle’s medium (DMEM; Gibco, USA) containing 20% fetal bovine serum (FBS; Gibco) and 100 U/ml penicillin and 100 U/ml streptomycin (Thermo USA). The cells were incubated in an incubator at 37 °C and 5% CO_2_. When the fibroblasts grow to the 4th to 7th generations, the cells exponentially growing at this time will be used for cell experiments.

### ART treatment

ART was provided by Li Shizhen Pharmaceutical Co., Ltd. (Hubei, China). Fibroblasts were cultured in 6-well plates, 96-well plates, or 10-cm-diameter culture dishes. GSK2606414, a PERK signaling pathway inhibitor, was purchased from Medchem Express Co., Ltd. (Shanghai, China). Q-VD-Oph, a caspase signaling pathway inhibitor, was purchased from Medchem Express Co., Ltd. (Shanghai, China). When the cells were grown to nearly 80%, they were rinsed with phosphate buffer saline (PBS) for three times. Human fibroblasts were treated with ART. The control group was incubated with PBS. Subsequently, experimental and control cells were collected for subsequent cell experiments.

### Cell viability assay

The Cell Count Kit-8 (CCK-8, Dojindo, Tokyo, Japan) was employed for testing fibroblast viability. The fifth to seventh generations of fibroblasts were taken, and the cells were planted in two 96-well plates. In one group, when the cells are grown to about 80%, the cells will be treated with ART at different concentrations (0, 5 μM, 10 μM, 20 μM, 40 μM, and 80 μM). Twenty-four hours later, the metabolites were first removed through PBS buffer washing, and then DMEM 100 μl and 10 μl CCK-8 were added to further culture the cells for 2 h. In the other group, when grown to 80%, the cells will be treated with 10 μM for 0, 12, 24, 36, 48, 60, and 72 h. Their optical densities were measured at 450 nm using a Microplate absorbance reader (Bio-Tek, Elx 800, USA). Cell viability was calculated based on the reference manual.

### Cell apoptosis was analyzed by flow cytometry

Viable cells were collected and planted in 6-well plates, and then the cells were placed in the incubator overnight. Three well cells in a 6-well plate were classified as a control group, and the remaining were cultured for 12 h in the incubator by being treated with 10 μM. Then, the cells were harvested and rinsed three times with an appropriate amount of PBS buffer solution. To grow suspension cells, the cells were suspended in 1 ml binding buffer× 1, and 100 μl of cell suspension was transferred to a test tube. Subsequently, 5 μl FITC Annexin V and 5 μl PI were added to each tube. Finally, a 400 μl 1× binding buffer was added to a test tube, and the cells were stored at ambient temperature in a light proof way. The mixture containing FITC Annexin V and PI was incubated for 15–20 min, and the apoptosis of the mixture was finally detected by flow cytometry.

### Western blot analysis

Different concentrations of ART treated cells and cells of the same concentration of ART were harvested for different treatment times to conduct Western blot analysis. First, the collected fibroblasts were treated on ice with a radioimmune-deposition (RIPA) buffer (Beyotime, Hangzhou, China). Subsequently, sonicate and centrifuge processing were performed to harvest proteins. Lastly, the concentration of protein was detected using BCA Protein Assay Kit (Beyotime, Hangzhou, China). Further, 40 μg of each protein lysate were separated on the Western blot at a 5–12% sodium dodecyl sulphate-polyacrylamide gel electrophoresis (SDS-PAGE) and then transferred to polyvinylidene difluoride (PVDF) membranes (Millipore, Bedford, MA) for 1.5 h at 200 mA. Next, the PDVF membrane was blocked for 2 h at ambient temperature with 5% skimmed milk. Then, PVDF membrane was washed with tris-buffered saline and Tween20 (TBST) solution. Following the reference manual, PVDF membranes were incubated with primary and secondary antibodies continuously. The primary antibodies were used, including anti-cleaved-poly ADP-ribose polymerase (cleaved PARP), anti-cleaved caspase3, anti-Bax, anti-Bcl2, anti-78 kDa glucose-regulated protein (GRP78), anti-CHOP, anti-PERK, anti-phospho-PERK (p-PERK), anti-eukaryotic translation initiation factor 2α (eIF2α), anti-phospho-eIF2α (p-eIF2α), and anti-glyceraldehyde-3-phosphate dehydrogenase (GAPDH) and secondary antibodies including antimouse or antirabbit IgG. All of the above antibodies were purchased from Cell Signaling Technology (Beverly, MA, USA); the concentration of the antibodies used in this study was 1 μg/ml.

### TUNEL assay in fibroblasts

Tunnel staining was employed to test apoptosis of human fibroblasts (KeyGEN, Nanjing, China). The experimental methods rigorously followed the manufacturer’s instructions. Fluorescence microscope was used to observe the features of apoptosis. Fibroblasts stained by TUNEL were considered apoptotic. The total number of fibroblasts was calculated using DAPI staining.

### Animals

The animal experiment was approved by the Animal Ethics Committee of Yangzhou University, and all the rabbits received humanitarian care. Twenty-four mature 4-month-old male New Zealand rabbits weighed 3.8 kg for this study. Rabbits were split into four groups at random (six in each group), including ART (15 mg/kg) group, (30 mg/kg) group, (60 mg/kg) group, and control group. Before the experiment, rabbits were kept in captivity for 1 week to adapt to the laboratory environment.

### Local application of ART

An intraarticular adhesion animal model was built based on previous scholars’ studies [20]. New Zealand rabbits were first given an intravenous injection of 2% pentobarbital (1.5 ml/kg) to achieve general anesthesia. Subsequently, the hair around the knee joint was shaved, the exposed skin was disinfected with iodophor, and the femoral condyle was then exposed. The cortical bone of nearly 10 mm × 10 mm was removed on both sides of the femoral condyle, and the articular cartilage was protected carefully. After the operation area was disinfected and hemostasis, the wound would be sutured. Next, the skin disinfection in the surgical area was achieved, and local injection concentrations of 15, 30, and 60 mg/kg ART were applied in the corresponding experimental group. The control group was injected with an equal amount of physiological saline. The drug was injected once per 2 days for five times. Furthermore, after the surgery, the cefazolin sodium was intramuscularly injected for 3 days to prevent postoperative infection of the rabbit; the surgical knee joint was externally fixed for 4 weeks in the Kirkner wire bending position.

### Histological analysis

Histological analysis was conducted 4 weeks after surgery. One rabbit was taken from each group at random. Subsequently, the rabbits were euthanized, the knee joint was opened along the original incision, and then the knee joint was removed (including all connective tissue and fibrous adhesion scar). The samples were fixed in 10% formalin for 1 week and then decalcified for 2 weeks. The tissue was embedded in paraffin, and transverse sections 5 mm perpendicular to the femoral axis were removed and stained with hematoxylin-eosin (HE) and Masson’s trichrome, respectively. Intraarticular scar adhesion and intraarticular collagen tissue were further evaluated under a × 400 magnification of an optical microscope.

### Immunohistochemical staining

The expressions of GRP78 (10 μg/ml) and CHOP (10 μg/ml) of the sections were detected using immunohistochemical staining. After deparaffinization and rehydration in gradient ethanol, the sodium citrate was added to the distilled water to pretreat the sections. After sealing with 10% goat serum at ambient temperature for 30 min, these sections were incubated with anti-GRP78 antibody and anti-CHOP antibody in an environment of 4 °C. They were then incubated at ambient temperature for 2 h and then analyzed with a coating tool to detect antibody binding. Subsequently, the sections were stained with hematoxylin. Finally, these sections could be observed under an optical microscope.

### Statistical analysis

SPSS 19.0 statistical software was employed to analyze the data. The independent sample *t* test was used in our experiment. All data were expressed as the mean ± standard deviation (S.D) values. Experiments were repeated three times. Statistical significance was defined as *P* < 0.05.

## Results

All animals received care following the principles of Laboratory Animal Care of international recommendations. The experimental protocol was approved by the Animal Care and Research Committee of the Yangzhou University, China. There were no surface or deep infections and no complications from surgical incisions.

### Histological analysis of the effect of ART on the intraarticular scar adhesion

**Fig. 1:**
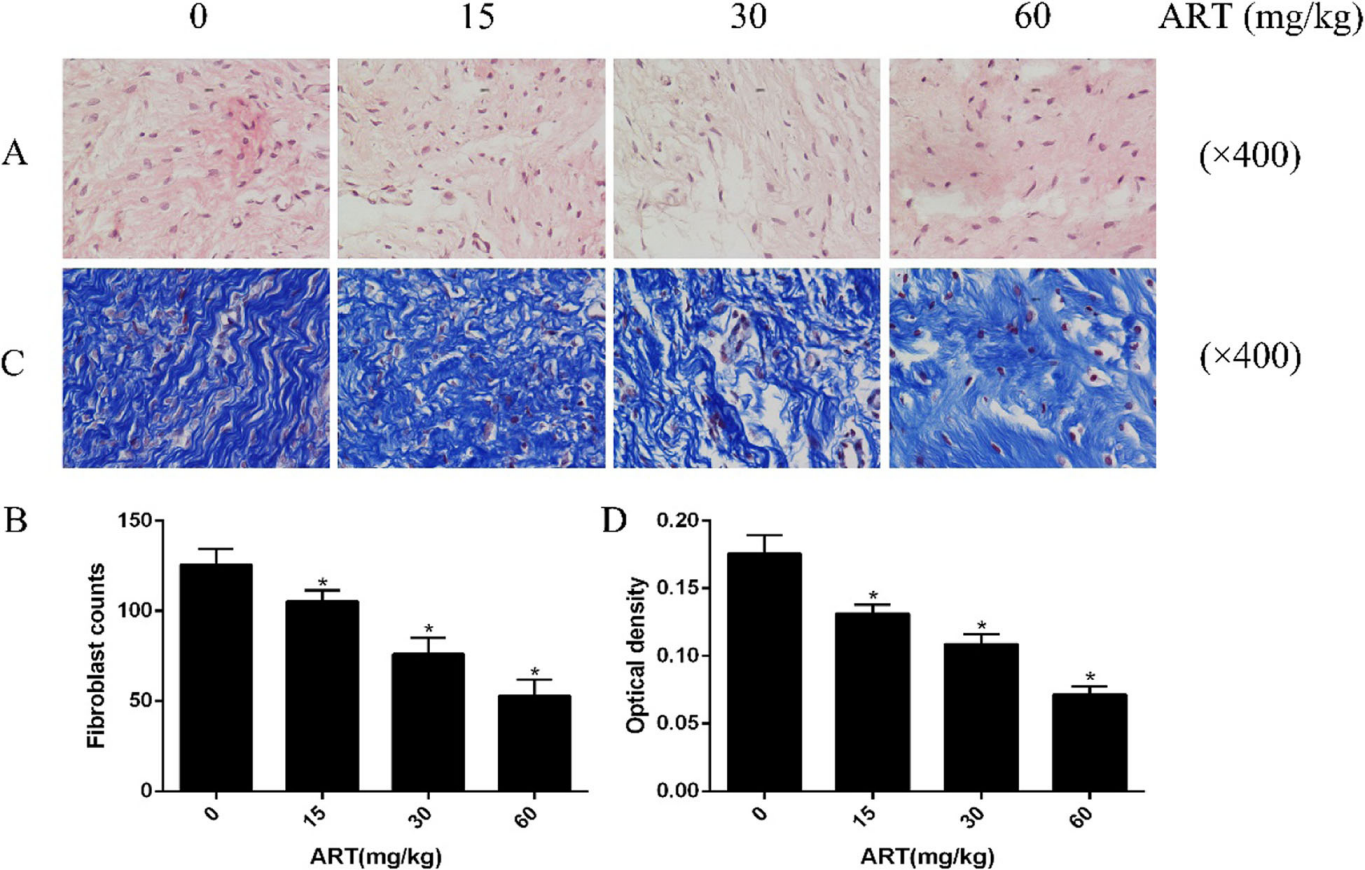
Histological analysis of the fibroblasts from the intraarticular scar tissue that had been treated with saline, 15 mg/kg, 30 mg/kg, and 60 mg/kg ART. **a** Dense scar tissue was observed in the operative areas treated with saline, 15 mg/kg, 30 mg/kg, and 60 mg/kg ART. **b** The number of fibroblasts were decreased as the concentration of ART increased. The sections were stained with HE, and the magnification is × 400. *P < 0.05 versus control group. **c** The collagen tissues are blue in the Masson’s trichrome-stained sections under the light microscope (× 400). **d** The results of Masson were expressed as optical density and showed in the histogram. *P < 0.05 versus control group

Several important information can be acquired from HE staining images. In the experimental group, the scar tissues of 15 mg/kg, 30 mg/kg, and 60 mg/kg was less than those of the control group, and the 60 mg/kg group was still lower than the 15 mg/kg and 30 mg/kg group (Fig. 1a). The number of fibroblasts was measured by image pro plus, and it was found that the number of fibroblasts gradually decreased as the concentration of ART rose (Fig. 1b). Masson’s trichrome staining image displayed that, compared with the control group, collagen density in the experimental group was significantly lower than the control group. The trend is similar to that of HE staining (Fig. 1c, d). Based on the above results, we can preliminarily conclude that local application of ART can reduce the scar adhesion in the joint.

### ART inhibits intraarticular adhesion through ERS

**Fig. 2:**
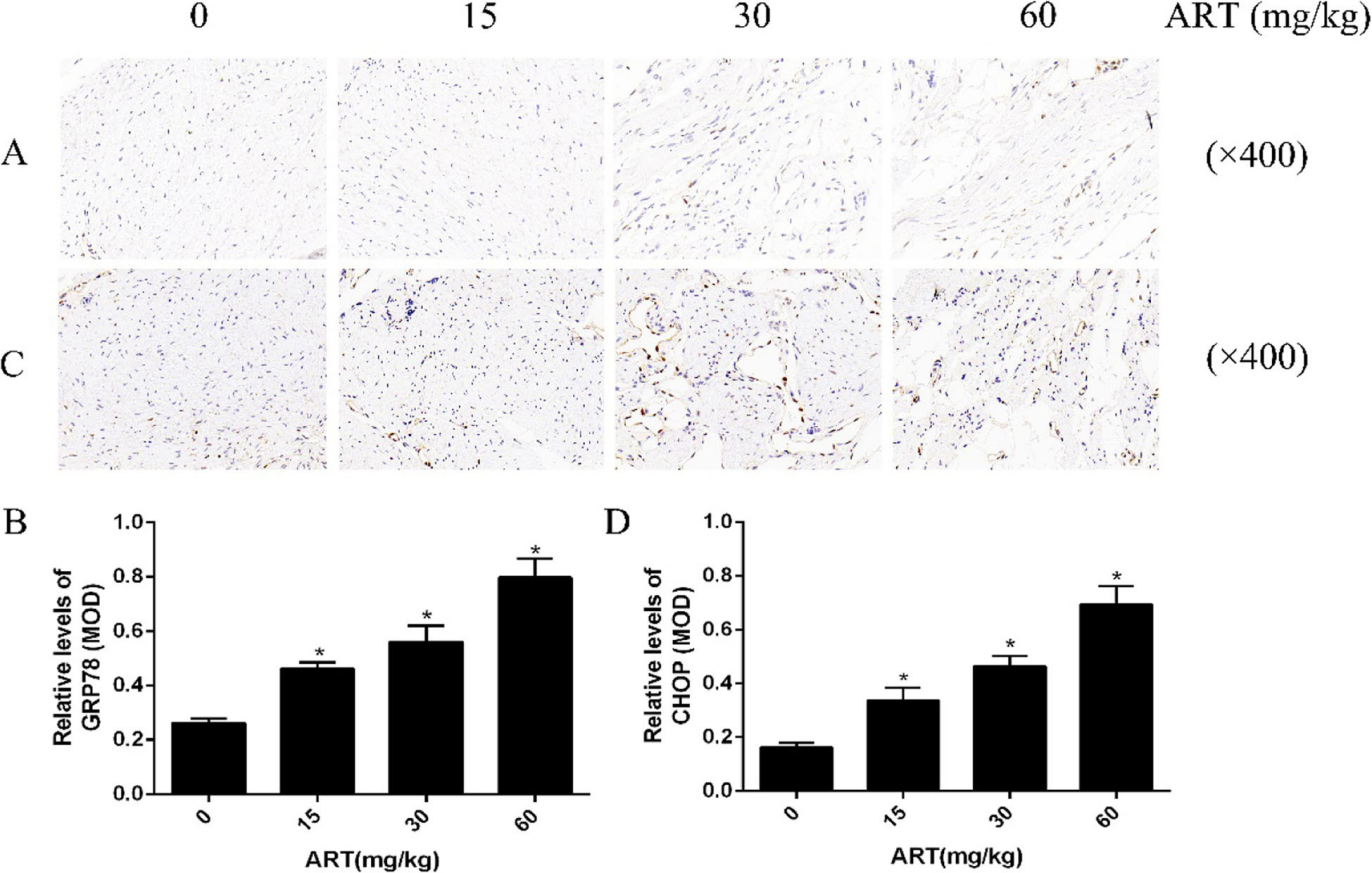
The expression of GRP78 and CHOP in the ART-treated intraarticular scar tissue. Immunohistochemical analysis of GRP78 (**a**) and CHOP (**c**) in the intraarticular scar tissue treated with saline, 15 mg/kg, 30 mg/kg, and 60 mg/kg ART. The results of GRP78 (**b**) and CHOP (**d**) expression were expressed as mean OD and showed in the histogram. *P < 0.05 versus control group

In order to further explore the mechanism, we examined the expression of markers GRP78 and CHOP by immunohistochemical analysis. Immunohistochemical analysis and the results of quantitative analysis of immunohistochemistry showed that GRP78 and CHOP in the cytoplasm of fibroblasts in the ART-treated group were significantly higher than those in the control group which showed a concentration-dependent manner (Fig. 2a–d). We can conclude that ERS was involved in ART-mediated delayed intraarticular adhesion.

### Effect of ART on fibroblasts

**Fig. 3:**
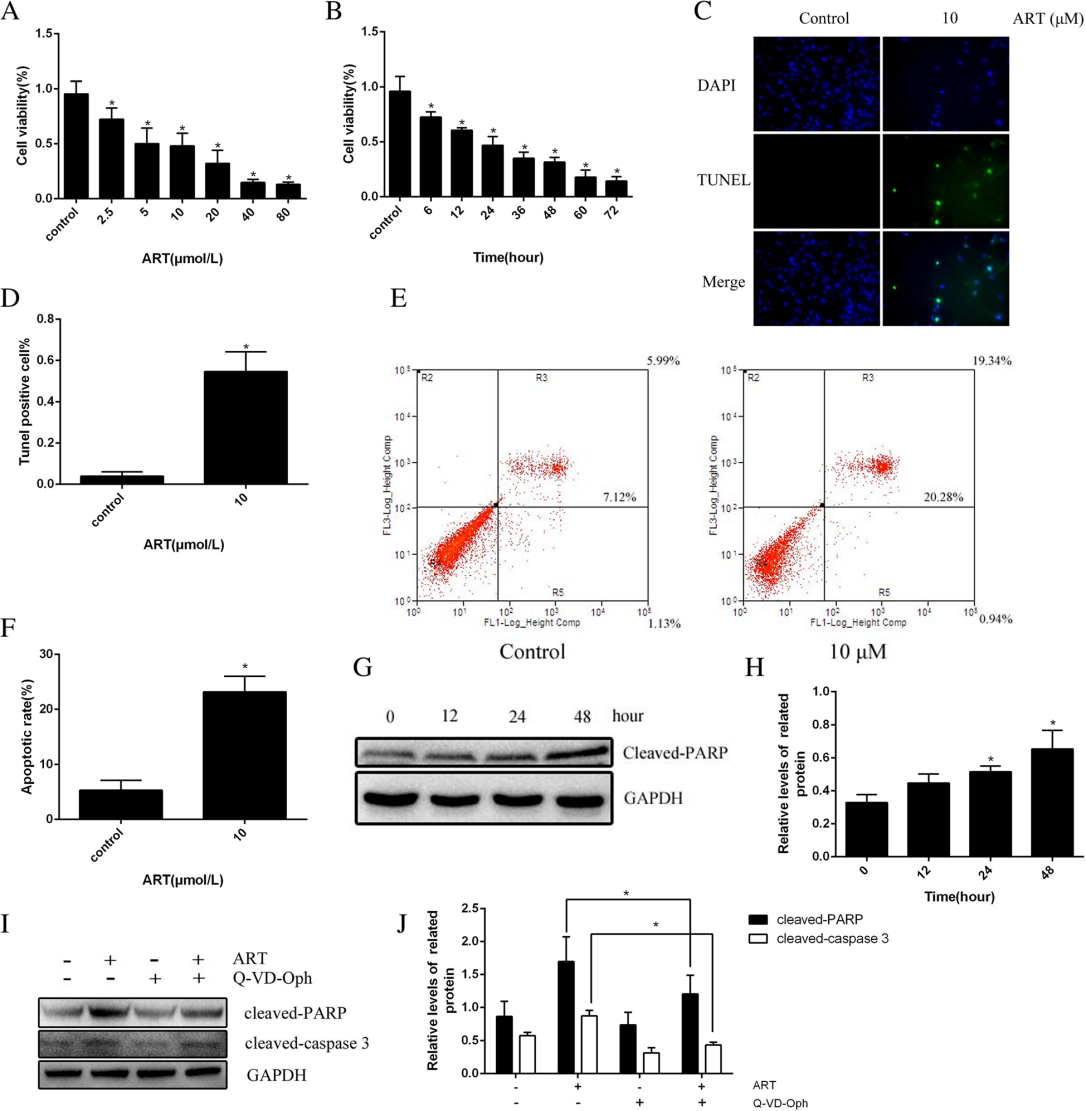
ART caused fibroblast apoptosis. **a** Dose-dependent effects of ART on fibroblasts after an overnight treatment with different concentrations of ART. **b** Time-dependent effects of 10 μM ART on fibroblasts following treatment for various durations; viability was determined by the CCK-8 assay. **c**, **d** The ART-treated cells were stained with Tunnel. **e**, **f** The apoptotic cells were stained with Annexin V/PI dual-staining after treatment with 10 μM ART. **g**, **h** The Western blot revealed that ART induces the expression of cleaved-PARP. **i**, **j** The Western blot revealed that the cytoprotective effect of caspase inhibitors. GAPDH was included as a control. *P < 0.05 versus control group

Fibroblasts treated with different concentrations of ART and cultured with the same concentration of ART for different time were detected by cck-8 method. It was found that ART could inhibit the viability of fibroblasts in a concentration-dependent and time-dependent manner (Fig. 3a, b). To determine whether ART affects cell viability through apoptosis, we use tunnel staining and flow cytometry, and the results showed that ART could induce the apoptosis of fibroblasts (Fig. 3c–f). In addition, cleaved-PARP were used as apoptosis marker proteins; Western blot further showed that ART can induce fibroblast apoptosis (Fig. 3g, h). We use Q-VD-OPh, a caspase signaling pathway inhibitor, which demonstrated the cytoprotective effect of caspase inhibitors by Western blot quantitative analysis (Fig. 3i, j). In summary, ART can reduce cell viability through apoptosis.

### ART can induce fibroblast apoptosis through PERK pathway

**Fig. 4:**
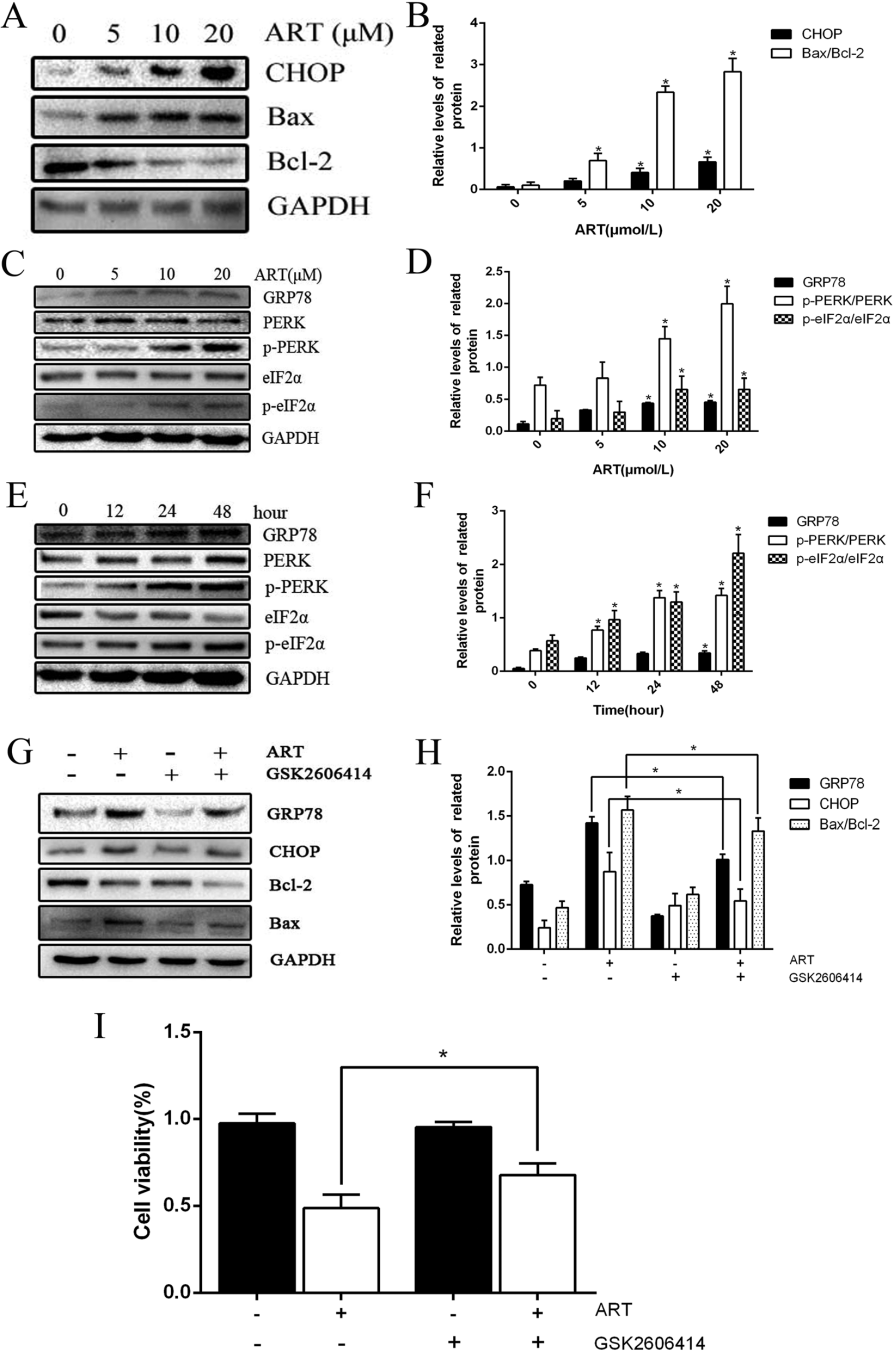
ART induced ER stress in the fibroblasts. **a**, **b** The expression of CHOP, Bcl-2, and Bax in cells that had been treated with 0, 5 μM, 10 μM, 20 μM ART overnight was determined by western blot analysis. GAPDH was included as a control. **c**–**f** The dose- and time-dependent effects of ART on the expression of proteins associated with the PERK signaling pathway, including p-PERK, PERK, p-eIF2α, and eIF2α, were determined by western blot analysis. GAPDH was used as a control. **g**, **h** After treated with or without GSK2606414, the fibroblasts were treated with 10 μM ART for 24 h, the detected by Western blotting with antibodies specific for GRP78, CHOP, Bax, Bcl-2, and GAPDH. **i** After treated with or without GSK2606414, the fibroblasts were treated with ART as described before and then cell viability was measured. *P < 0.05 versus control group

After different concentrations of ART treatment, it was found that the proapoptotic proteins CHOP and Bax were upregulated in expression, while the anti-apoptotic Bcl-2 expression was downregulated, and the ratio of Bax/Bcl-2 increased (Fig. 4a, b). Figure 4c, d showed that GRP78, the ratio of p-PERK/PERK and p-eIF2α/eIF2α also showed an increasing trend and the effect is concentration-dependent. On the other hand, we treated fibroblasts with ART at a concentration of 10 μM for 0 h, 12 h, 24 h, and 48 h. The results showed that PERK pathway-related proteins were activated and gradually increased in a time-dependent manner (Fig. 4e, f). To further investigate the role of PERK signal pathway in ART-induced fibroblast apoptosis, we use the PERK signal pathway inhibitor GSK2606414. The results showed that after the PERK pathway was inhibited, the expression of CHOP, GRP78, and Bax/Bcl-2 was decreased (Fig. 4g, h). CCK-8 results were similar (Fig. 4i); it was reported that after the inhibition of PERK pathway, the cell viability was higher than that of the normal drug treatment group. These results indicate that the ART could induce fibroblast apoptosis by activating the PERK signal pathway.

## Discussion

ART is a derivative of artemisinin, which refers to a new structural antimalarial drug characterized by high efficiency, quick effect, low toxicity, and low tolerance. It has been adopted for mild to severe basic treatment for malaria infection. A growing number of studies have suggested that ART and its activated metabolite dihydroartemisinin exert pharmacological effects on anti-tumor, anti-inflammatory, anti-sepsis, anti-angiogenesis, anti-fibrosis, as well as immune regulation [9–12]. This study aimed to gain insights into the anti-fibrosis of ART.

Knee joint adhesion refers to a serious complication caused by knee surgery or knee injury. The clinical symptoms are largely limited by the range of knee joint activity and chronic joint pain; it eventually causes various sequelae (e.g., articular cartilage degeneration and knee joint stiffness), resulting in surgical failure. Existing studies considered that the major pathogenesis of knee adhesions includes [21, 22] (1) trauma/surgery caused early local soft tissue hemorrhage, activated inflammatory response, causing fibrin and inflammatory cell exudation; (2) fibrinolysis and the balance of the antifibrinolytic system are broken, causing fibrin deposition, and stimulating the migration, the proliferation, as well as the secretion of extracellular matrix (e.g., collagen and fibronectin), which eventually leads to the accumulation of granulation tissue around the synovial membrane and joint capsule; and (3) collagen fibers are thickened, and microvascular closure disappear, leading to in granulation tissue fibrosis, synovial membrane, and joint capsule atrophy, which results in the reduction of space in the joint cavity and the reduction of hyaluronic acid secretion, the formation of intraarticular adhesions, and joints degenerative lesions of cartilage.

This study was conducted to investigate the role and mechanism of ART-induced human fibroblasts in the prevention of intraarticular scar adhesion. In vivo experiments, HE staining results suggested that the fibroblast counts in the 60 mg/kg ART group were significantly lower than those in the ART low concentration group and the control group, and were drug concentration dependent. As the concentration of ART rises, the results of Masson staining and HE staining are consistent. Masson staining showed that the density of collagen fibers was lower in the 60 mg/kg ART group than in the low concentration group and the control group, and it was concentration-dependent. In vitro, ART-treated human fibroblasts and tested by cck-8 showed that ART can reduce cell viability and promote fibroblast apoptosis in a concentration- and time-dependent manner. On the other hand, results of tunnel staining, flow cytometry, and increase of cleaved-PARP expression in ART-treated fibroblasts also indicates the apoptosis of fibroblasts. The above experiments in vitro and in vivo showed that ART can promote the apoptosis of fibroblasts and reduce the production of collagen fibers.

ER is a multifunctional organelle that plays an important role in maintaining the stability of the intracellular environment. After ERS occurs, the cells initiate an UPR to restore the homeostasis of the cells [23]. Under long-term severe ERS, UPR no longer exerted cytoprotective effects but started to exert cytotoxic effects (including apoptosis). Some scholars reported that the use of ingenuity pathway analysis (IPA) found that UPR is a crucial way to induce ERS by ART [24]. The PERK pathway is one of the classic ERS pathways. In the present study, upregulated expression of GRP78, CHOP, and Bax in fibroblasts after ART treatment, and the expression of Bcl-2 was downregulated, suggesting that ERS is activated. Furthermore, after ART treatment, the proteins (e.g., p-PERK and p-eIF2α increased), and the ratios of p-PERK to PERK and p-eIF2α to eIF2α in the PERK pathway were also upregulated, which was also concentration- and time-dependent, suggesting that the PERK pathway is involved in ART-mediated apoptosis. Furthermore, after treatment with GSK2606414, we found that the increasing trend of CHOP, GRP78, and Bax/bcl-2 was inhibited. Therefore, these results indicate that ART induces fibroblast apoptosis may be through the PERK pathway involved in the process of apoptosis.

## Conclusion

In conclusion, this preliminary study revealed that topical injection of ART could prevent joint scar adhesion. The possible mechanism may be the apoptosis of fibroblasts induced by PERK signaling pathway. Nevertheless, more experiments are required to verify the safety, efficacy, and toxicity of ART.

## Data Availability

The datasets supporting the conclusions of this article are included within the article and its supplementary materials.

## Abbreviations

ART: Artesunate
CCK-8: Cell counting kit-8
CHOP: C/EBP homologous protein
DMEM: Dulbecco’s modified Eagle’s medium
eIF2α: Eukaryotic initiation factor 2α
ERS: Endoplasmic reticulum stress
FBS: Fetal bovine serum
GAPDH: Glyceraldehyde-3-phosphate dehydrogenase
GRP78: 78 kDa glucose-regulated protein 78
HE: Hematoxylin-eosin
PARP: Poly ADP-ribose polymerase
PBS: Phosphate buffer saline
PERK: PRKR-like ER kinase
PVDF: Polyvinylidene difluoride
RIPA: Radioimmune-deposition
SDS-PAGE: Sodium dodecyl sulphate-polyacrylamide gel electrophoresis
TBST: Tris-buffered saline and Tween20
UPR: Unfolded protein response
